# Evaluating the diagnostic performance of miLab™ for detection of malaria parasites using nPCR as reference standard

**DOI:** 10.1186/s12936-026-05801-7

**Published:** 2026-02-12

**Authors:** Ebenezer Kojo Addae, Theophilus Awortwe-Quaicoe, Benedict Sackey, Richard Owusu Ansah, Richard Larbi, Kinako Denis Elia Dazangapai, James Opoku Frimpong, Thelma Owusuaa Ofori Amoako, Alexander Asamoah, Nana Ayisi-Boateng, Bernard Nkrumah, Franklin Asiedu Bekoe, Michael Owusu

**Affiliations:** 1 Centre for Health System Strengthening, Kumasi, Ghana; 2 Department of Medical Diagnostics, Faculty of Allied Health Sciences, KNUST, Kumasi, Ghana; 3 African Field Epidemiology Network (AFENET), Accra, Ghana; 4 Genomic and Infectious Disease Laboratory, KNUST, Kumasi, Ghana; 5 National Malaria Elimination Programme, Ghana Health Service, Accra, Ghana; 6 Department of Medicine, Kwame Nkrumah University of Science and Technology, Kumasi, Ghana; 7 Public Health Division, Ghana Health Service, Accra, Ghana

**Keywords:** Malaria diagnosis, AI-assisted device, Automated microscopy, MiLab^™^, nPCR, Ashanti region, Ghana

## Abstract

Malaria remains a leading health threat in sub-Saharan Africa, causing over 600,000 deaths annually. Ghana, ranked among the top 15 in malaria burden in Africa, relies heavily on microscopy for diagnosis due to its affordability and established use. However, limitations in sensitivity, turnaround time, and availability of skilled personnel despite ongoing national training efforts stress the need for improved diagnostics. This study evaluated miLab^™^, an AI-assisted automated malaria detection platform, using nested PCR (nPCR) as the reference standard. We conducted a hospital-based cross-sectional study from August 2024 to June 2025 in three malaria-endemic communities in Kumasi, Ashanti region and enrolled 300 suspected malaria patients (168 females, 132 males; aged 1–87 years, median 24). Blood samples were analyzed independently by miLab^™^, two independent mid-level microscopists, and nPCR. Discrepancies between the two microscopists were resolved by a WHO expert microscopist (adjudicated microscopy) to establish a microscopy reference standard. Diagnostic accuracy, correlation and measurement agreement were determined using GraphPad Prism Version 8. Parasite densities estimated by miLab^™^ ranged from 0.95 to 5.34 log parasites/µL (median 3.52, IQR 2.93–3.91). For mid-level microscopist 1, densities ranged from 2.02 to 5.43, (median 4.02, IQR: 3.22–4.50), while Mid-level microscopist 2 measured densities between 1.98 and 5.22, (median 3.78, IQR: 3.15–4.23). When compared to nPCR, miLab^™^ demonstrated a sensitivity of 94.23%, specificity of 98.98%, and accuracy of 97.33%, while adjudicated microscopy showed a sensitivity of 85.58%, specificity of 97.96%, and accuracy of 93.67%. These findings indicate that miLab^™^ has better performance compared to microscopy for detection of malaria parasites. miLab™ could offer a reliable, rapid diagnostic alternative suitable for malaria-endemic, resource-limited settings where timely and accurate diagnosis is critical for effective case management and control.

**Supplementary Information:**

The online version contains supplementary material available at 10.1186/s12936-026-05801-7.

## Introduction

Malaria is a complex, non‑specific, multi‑organ disease with frequent fatalities, causing significant mortality, morbidity, and socio-economic disruption [1]. According to the WHO World Malaria Report 2025, an estimated 282 million malaria cases occurred globally in 2024, with approximately 610,000 deaths. Sub-Saharan Africa accounted for 94% of all cases and 95% of all malaria-related deaths, with children under 5 years of age constituting the majority of fatalities [2, 3]. In 2020, malaria exposure was reported in 11.6 million out of 33.8 million pregnancies, as the second most vulnerable group [4].

Ghana ranks among the top 15 malaria-endemic countries in Africa, contributing about 2.5% of the global malaria burden [2]. It has been indicated that *Anopheles gambiae* sensu stricto, which is a more efficient vector compared to *Anopheles arabiensis* and *Anopheles funestus*, is the most predominant species in the middle belt of Ghana [4, 5]. Recently, *Anopheles stephensi* has been reported to be contributing to the transmission of cases of malaria [6]. Three out of the five known *Plasmodium* parasites have been reported to cause malaria in Ghana [4, 7]. These are *Plasmodium falciparum* mono-infections (97%), *Plasmodium malaria*e mono-infections (1.0%), and *Plasmodium ovale* mono-infections (1.6%). *Plasmodium vivax* and *Plasmodium knowlesi* have not yet been identified in blood films in Ghana [7]. Mixed plasmodium infections have also been published [8, 9]. A prevalence of 13.3% mixed infection of *Plasmodium falciparum* with either *Plasmodium malariae* or *Plasmodium ovale* cases in the eastern part of the country [8] and 20.9% *Plasmodium falciparum* mono-infections have been reported [10].

Malaria diagnosis, both clinical and laboratory, is paramount to improving patient health outcomes. According to the World Health Organization, laboratory diagnostic tests for malaria are expected to achieve high accuracy in detecting *Plasmodium* parasites at the species level, quantify parasite density to guide clinical management, and demonstrate sufficient sensitivity to detect low-level parasitemia [11]. They should also reliably confirm the presence or absence of infection and support monitoring of treatment response, particularly in the context of antimalarial resistance surveillance. These criteria align with the broader call for diagnostics that not only support clinical decision-making but also strengthen epidemiological surveillance [12]. Over several decades, the laboratory diagnosis of malaria has undergone significant improvements with the introduction of innovative methodologies such as Rapid Diagnostic Tests (RDTs) and Polymerase Chain Reaction (PCR). Conventional PCR, while generally more reliable than RDTs, is limited by its bulky equipment, necessity for technical expertise, and variable sensitivity depending on assay design [11, 13]. To this end, conventional microscopy remains the gold standard in the laboratory diagnosis of malaria [13, 14], even though it has several inherent challenges. These include the technical expertise of the microscopists, the quality of staining of blood smears, the quality of the microscopes used, cost of training microscopists (especially in resource-limited settings), turnaround time of the test procedure, and limitation in detecting very low parasite levels [15, 16]. These challenges have led to the invention of automated analyzers, which can analyze blood samples to identify the presence of malaria parasites and to quantify parasites if present.

Artificial Intelligence (AI) analytics has gained significant attention in recent years due to its broad applicability and potential for continuous improvement through machine learning [17]. AI systems can analyze vast amounts of data, recognize patterns, and make decisions with minimal human intervention. In the scope of malaria diagnosis, digital image recognition using AI algorithms have been explored to complement the human element in blood film interpretation, particularly on thin films [17–19]. Various automated-microscopy malaria laboratory diagnostic systems have incorporated AI with automated Romanowsky staining techniques to reduce variability in film preparation [17, 20–24], with sensitivities (Sn) and specificities (Sp) ≥ 75%. These automated systems have been evaluated in different countries across the globe, including Thailand [23], Netherlands [20], Korea [21], India and Kenya [24], Equatorial Guinea [25], Peru [17] and Ethiopia and Ghana [22].

Our Centre, Center for Health System Strengthening (CfHSS) collaborated with the Public Health Division of the Ghana Health Service (GHS) and other partners to evaluate the performance of an AI-integrated, fully automated digital microscope Point-of-Care (POC) device for malaria parasite detection using nested PCR (nPCR) as the reference standard.

## Methodology

### Study design and sample collection

**Fig. 1:**
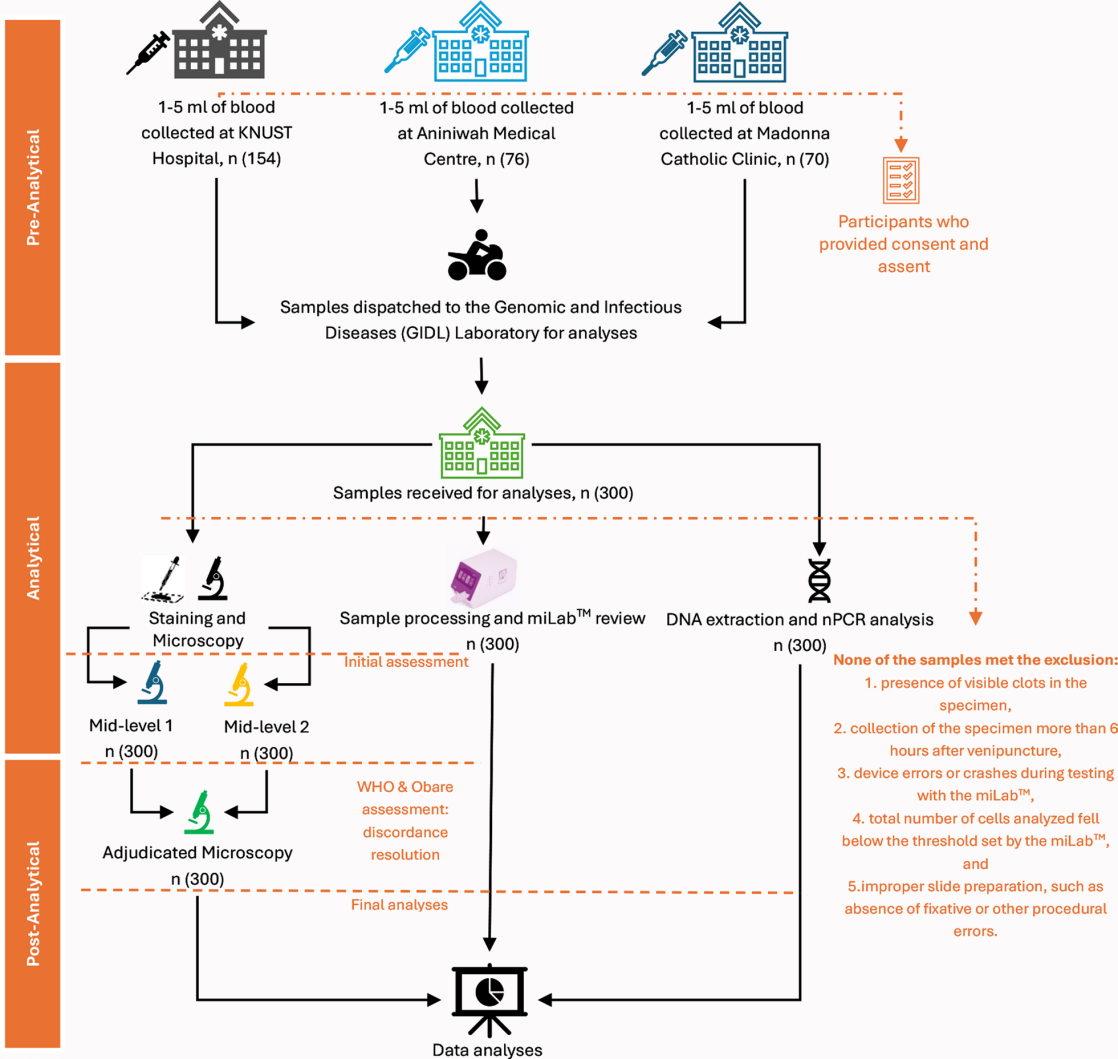
Flowchart of study design and sample collection

This hospital-based cross-sectional study was conducted from August 2024 to June 2025 in selected malaria-endemic communities in Kumasi, Ashanti region, Ghana. Febrile patients of any age who were clinically suspected of malaria and referred for routine malaria testing were enrolled. Trained facility laboratory staff drew 1–5 ml of venous blood into EDTA-K2 tubes after consent/accent was provided by patients. Thick and thin films were prepared onsite using the WHO malaria slide preparation template [26]. Whole blood samples and blood films were transported from healthcare facilities to the Genomic and Infectious Disease Laboratory (GIDL) at the Kwame Nkrumah University of Science and Technology (KNUST) within 6 h at room temperature. Film staining and whole blood processing were subsequently performed. All samples were evaluated under standardized protocols at GIDL using miLab^™^ (Noul Co., South Korea), microscopy and nPCR which was the reference standard. For microscopy, slides were examined by two independent mid-level (routine) microscopists in a blinded manner. Discrepancies between the two microscopists were resolved by a third WHO expert microscopist according to the WHO three-reader method [27]. The miLab^™^ device, was used for automated detection, species identification, and quantification of malaria parasites. nPCR was conducted following standard protocols for genus and species-level detection of *Plasmodium* spp., including appropriate positive and negative controls. All analyses were performed blinded to the results of the other methods. A schematic overview of the full diagnostic workflow is provided in Fig. 1.

### Inclusion and exclusion criteria

All febrile individuals of all ages who presented at the selected healthcare facilities clinically suspected of having malaria and willingly provided informed consent by the patient or their legal guardian in the case of minors were recruited for the study.

Individuals who had received antimalarial treatment within 48 h prior to sample collection, those with incomplete clinical or demographic records, patients from whom insufficient blood volume was obtained to perform all three diagnostic tests (microscopy, miLab^™^, and nPCR) and those who were unwilling to participate in the study were excluded.

### Study site

**Fig. 2:**
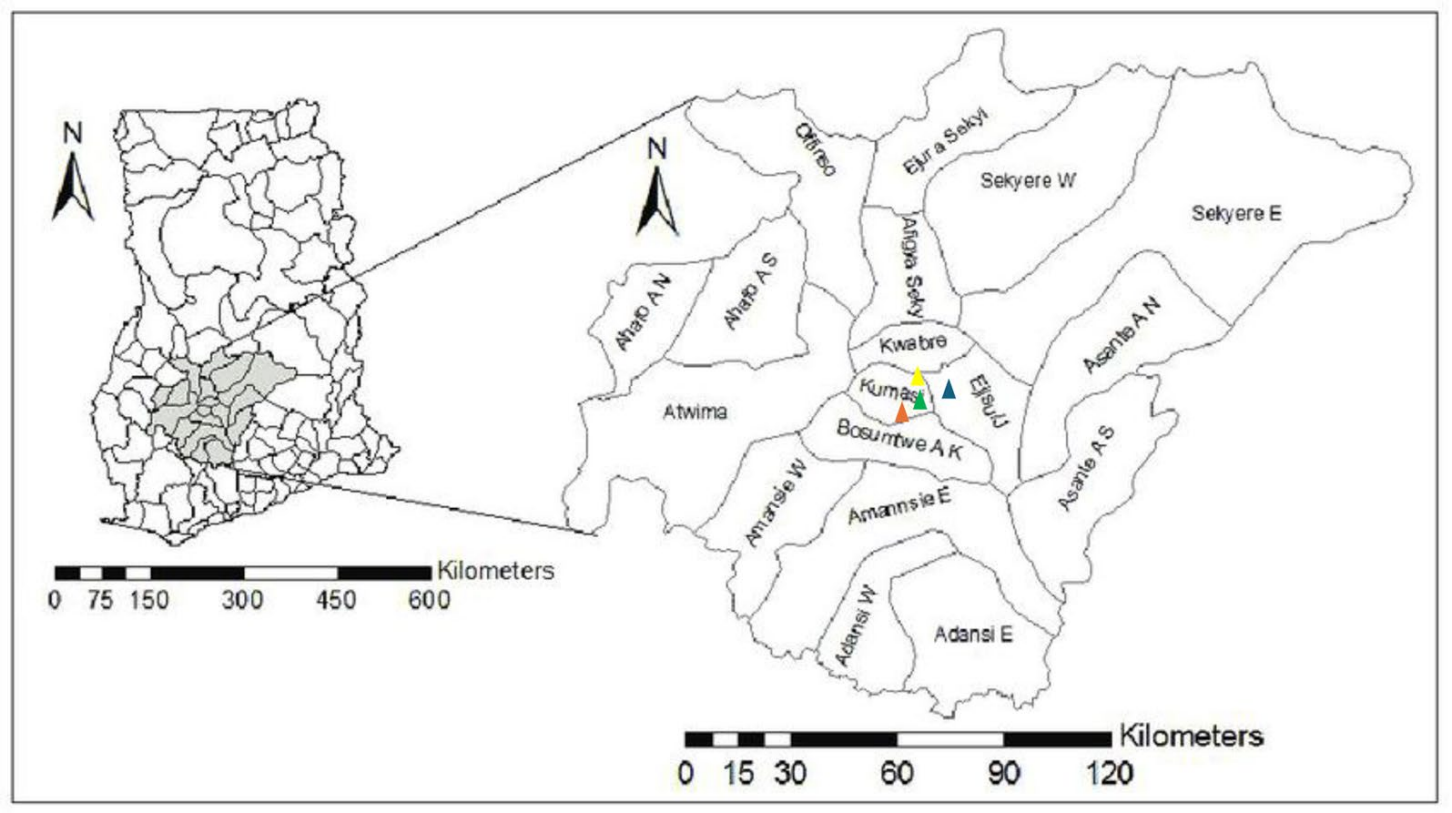
Map of study sites. The yellow triangle represents Aniniwah Medical Centre, orange represents KNUST Hospital, blue represents Madonna Catholic Clinic, and green represents GIDL. Map adapted from (Osei [28])

We enrolled patients from three healthcare facilities in the Ashanti Region of Ghana (Fig. 2): The University Hospital Services (KNUST Hospital) in Kumasi, Aniniwah Medical Centre in Emena, and Madonna Catholic Clinic in Ejisu. The KNUST Hospital is one of the largest referral facilities in the region and in addition serves a large surrounding population including Ayigya, Bomso, Ayeduase, Kotei, and Boadi. Aniniwah Medical Centre operates as a private diagnostic and fertility clinic offering round-the-clock outpatient, inpatient, laboratory, and imaging services to communities within the Kumasi metropolitan area. Madonna Catholic Clinic, functions as a primary care facility in Ejisu and plays a crucial role in malaria diagnosis and treatment within its peri-urban catchment.

Site selection was guided by population representativeness, proximity to the GIDL-KNUST to facilitate sample processing and analysis within the required six-hour window, resource availability, infrastructural adequacy, and ethical feasibility to ensure efficient participant recruitment. Participants were enrolled following a serial accrual approach, i.e., consecutively as they presented to the study site until the target sample size was achieved, ensuring a pragmatic representation of routine clinical cases.

### Sample size

A minimum sample size of 209 was estimated to evaluate the diagnostic performance of the miLab™ platform. This was calculated using a Type I error rate of 5% (α = 0.05), 90% expected sensitivity and specificity, and a marginal error of 10% [29]. With a *Plasmodium falciparum* mono-infection prevalence of 20.9% [10], the target sample size was projected to yield at least 60 malaria-positive cases. This number was deemed sufficient to preliminarily assess diagnostic accuracy against Go/No-Go thresholds set by the device manufacturer while balancing resource and field constraints.

### Laboratory procedures

#### Experimental data

Prior to the study implementation, a pilot evaluation was conducted using 30 blood samples collected from the same health facilities designated for the main study, and with the same staff who would be involved in full-scale implementation. This preliminary exercise aimed to validate field conditions, test the operational workflow of miLab^™^ alongside microscopy and nPCR, and ensure consistency in sample processing. The pilot also served to identify and address any potential procedural or logistical challenges in advance, allowing the research team to refine protocols and be better prepared to manage similar issues during the main study.

#### miLab^™^ equipment description

The miLab^™^ MAL is deployed as a sizable (~ 11 kg), bench-side, fully automated microscopy platform that can be positioned on a standard laboratory bench (Additional Fig. 1). In routine operation, each case was processed using a single cartridge, after which the platform carried out fully automated staining based on a modified Romanowsky protocol with solid-based Next-Generation Staining and Immunostaining (NGSI) stamping technology. Once prepared, the device actively scanned intact Red Blood Cells (RBCs) on thin smears, screening up to 300,000 RBCs per sample, and computed parasite density which spreads across all infection intensities: low (< 1,000 parasites/µL), moderate (1,000–9,999 parasites/µL), high (10,000 ≤ 100,000 parasites/µL), and severe or hyperparasitemia (≥ 100,000 parasites/µL or parasitemia ≥ 5%) [11]. In practice, the platform generates diagnostic results within 15–25 min, requiring less than two minutes of hands-on input and only a 5 μl blood sample per test (Additional Table 1).

miLab^™^ employs a cartridge that uses whole blood for *Plasmodium Spp*. Detection, while the SafeFix acts as a fixative to preserve the integrity of the blood sample. Inside the cartridge, a thin smear is automatically prepared on a slide that is stained using an improved Romanowsky stain, which is also imbedded in the cartridge. Noul has developed hydrogel staining aiming to improve the accuracy of whole blood analyses. Three types of hydrogen patches containing dye reagents (eosin, methylene blue/oxidized methylene blue, Azure B and buffer) are subsequently stamped onto the blood smears to stain blood cells. Essentially, when dye-containing hydrogels contact blood cells, the dyes are transferred to the cells for staining. The hydrogel staining generates a high-quality, easily reproducible blood smear samples within 4 min, reducing testing, manufacturing costs and biological waste. The process of hydrogel staining has been described earlier [30].

#### miLab^™^ testing process

**Fig. 3:**
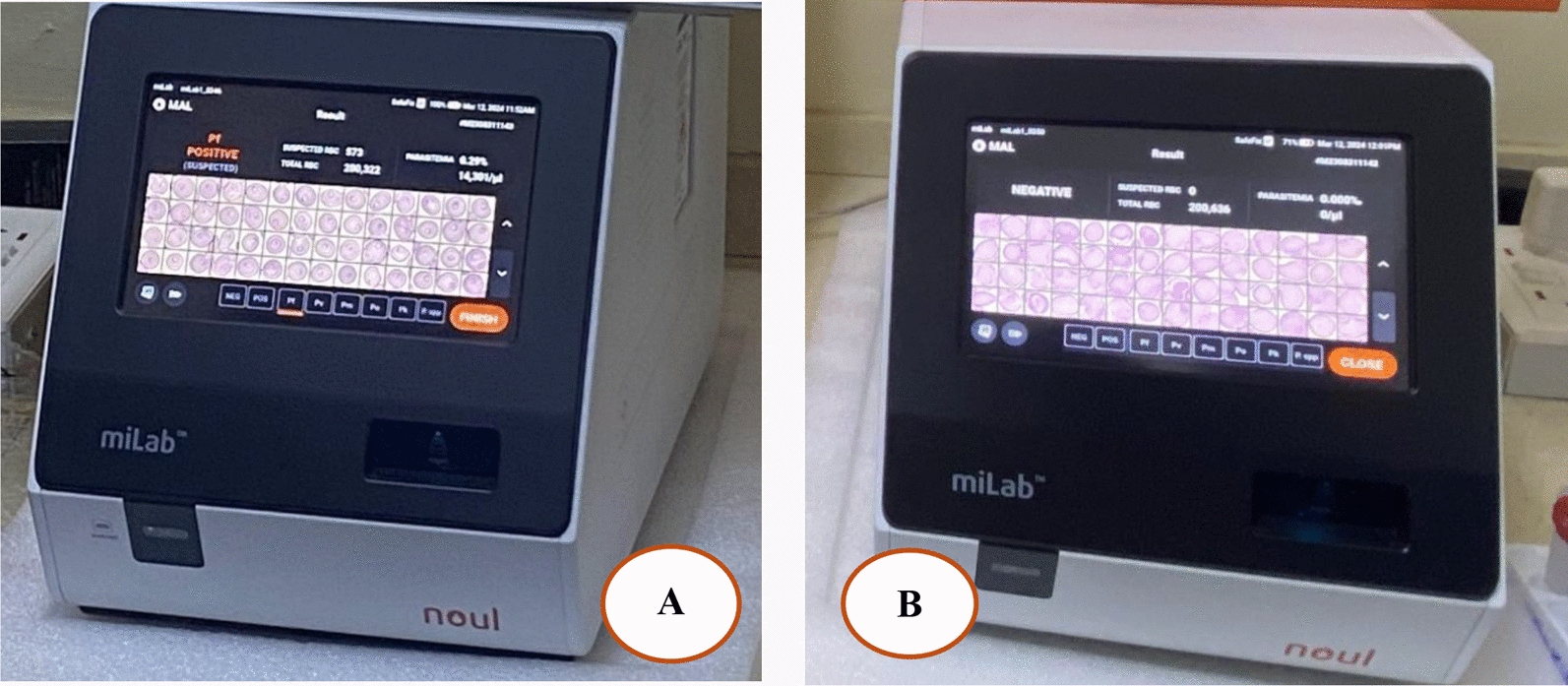
Digital display of **A** positive RBCs and **B** negative RBCs. Digital display of **A** positive RBCs and **B** negative RBCs. In **A** the miLab^™^ MAL platform identifies *Plasmodium falciparum* as “positive suspected,” with a suspected infected RBC count of 973, a parasitaemia estimate of 14,301/μL, and a total RBC count of 200,322. Image **B** shows a negative result with no parasites detected

**Fig. 4:**
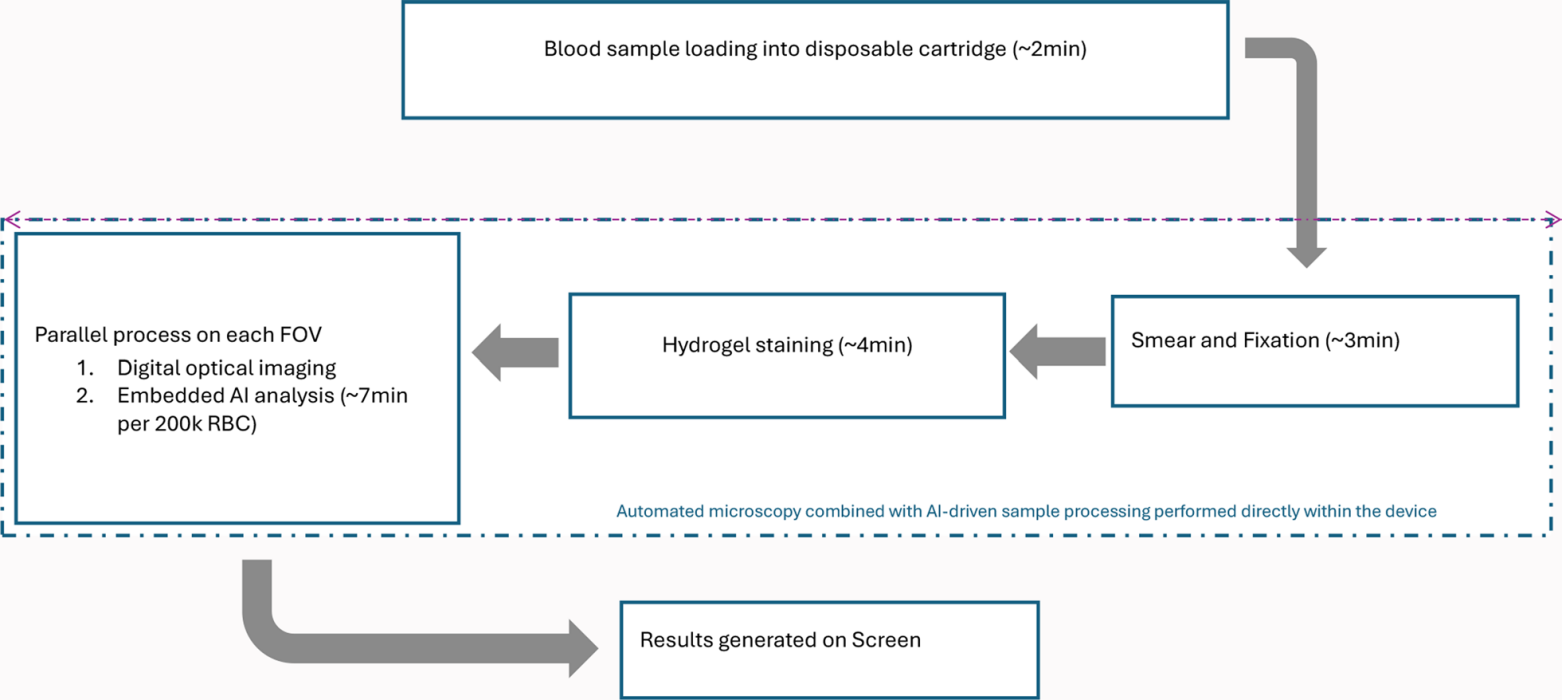
Workflow for miLab^™^ MAL sample processing. Blood is introduced into a disposable cartridge and fixed onto a polymer film. The system applies NGSI using hydrogel stamps. Stained films are imaged, and AI algorithms detect and quantify malaria parasites. Results, including parasitemia and infected RBC count, are generated within 15–25 min and displayed digitally

After staining is complete, the analyzer projects a digital picture of the smear, displaying all the cells in their preserved and stained architecture (Fig. 3). The analyzer then identifies the cells and provides a diagnostic summary of the patient’s smear. The reference for confirming miLab^™^ MAL results is the digital image result determined by expert microscopists using the miLab^™^ viewer program. The workflow is described in Fig. 4.

#### miLab^™^ parasite determination

Malaria parasite density was determined using pre-set calculations on the miLab^™^ instrument, which utilizes RBC count of up to 300,000; for this study, the default total RBC count of 200,000 was applied. The same absolute RBC value corresponding to thin films (5 million RBCs) was used. The miLab^™^ pre-set parasite count (Parasites/µL) was calculated using the formula:

$$\frac{Parasite}{{\mu L}}\, = \,\frac{Number\,of\,Rings\, + \,Number\,of\,Trophozoites}{{Total\,RBC\,Count}}\, \times \,5,000,000\,{{RBCs} \mathord{\left/ {\vphantom {{RBCs} {ml}}} \right. \kern-0pt} {ml}}$$

To reduce potential human bias in selecting high-density fields for parasite counting on thin films, the five Fields of View (FOVs) with the highest number of parasites were selected from the miLab^™^ analysis results. The counts of parasitized and total RBCs from these FOVs were used to calculate the parasite density using the same formula. The total RBC count included both parasitized and non-parasitized cells.

### DNA extraction

Genomic DNA was extracted using the QIAamp DNA Blood Mini Kit (Qiagen, Hilden, Germany) following the manufacturer's protocol with minor modifications. This extraction was optimized to ensure high-quality DNA suitable for downstream molecular analysis.

Briefly, 20 µL of Proteinase K was added to 200 µL of whole blood in a sterile 1.5 mL microcentrifuge tube. Cell lysis was achieved by adding 200 µL of Buffer AL, followed by incubation at 56 °C for 10 min. After incubation, 200 µL of absolute ethanol was added to precipitate the DNA. The mixture was transferred to a QIAamp spin column and centrifuged at 8,000 rpm for 1 min. The column was washed sequentially with 500 µL of Buffer AW1 and Buffer AW2 to remove proteins and other impurities by centrifugation at 8,000 rpm and 14,000 rpm, respectively. DNA was eluted in 60 µL of Buffer AE by centrifugation at 8,000 rpm for 1 min. The eluted DNA was stored at −20 °C for downstream PCR.

### DNA amplification by nPCR

**Fig. 5:**
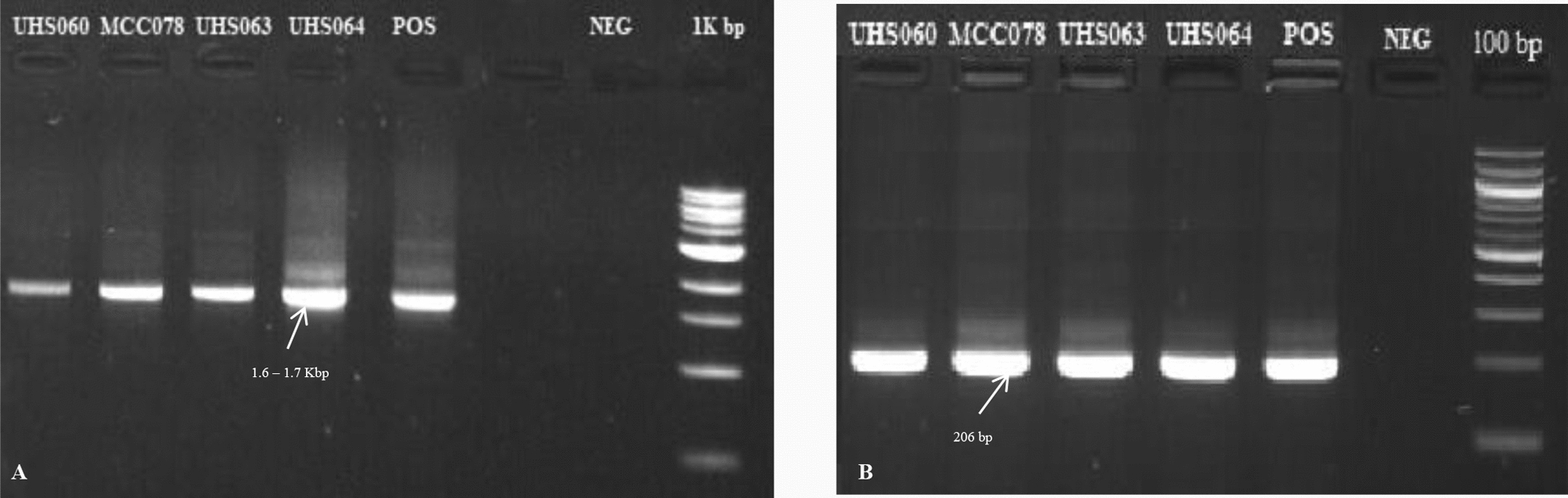
PCR amplicons on 1% agarose gel for genus-specific PCR products (**A**) and PCR amplicons on 2% agarose gel for species-specific PCR products (**B**). In panel **A**, the 1 Kbp marker represents the DNA ladder; POS denotes the positive control, and NEG indicates the negative control. In panel **B**, the 100 bp marker represents the DNA ladder; POS denotes the positive control, and NEG indicates the negative control

Nested PCR was used to detect *Plasmodium* genus and identify species-specific infections, following the established protocol by Snounou and Singh [16]. Amplification targeted the highly conserved 18S ribosomal RNA gene. The primary PCR (Nest 1, genus-specific; primers rPLU5 and rPLU6) was performed in a 25 µL reaction mixture comprising 5 µL of extracted DNA, 0.75 U of Taq DNA polymerase (0.15 µL), 2.5 µL of 10X PCR buffer, 2.0 µL of 50 mM MgCl₂, and 0.5 µL of 10 mM dNTPs. For the secondary PCR (Nest 2, species-specific), 1 µL of the primary PCR product served as template in a 25 µL reaction containing 0.75 U of Taq DNA polymerase (0.15 µL), 2.5 µL of 10X PCR buffer, 2.0 µL of 50 mM MgCl₂, 0.5 µL of 10 mM dNTPs, and 0.8 µL each of species-specific primers rFAL1 and rFAL2. The expected DNA products from both genus-specific and species-specific were assessed using a 1% and 2% agarose gel electrophoresis respectively (stained with SYBR Gel stain) (Fig. 5A, B).

The thermocycling conditions are provided in Tables 1, 2 whereas primer sequences used for amplification are provided in Table 3. Each run included a non-template control and a known positive control to validate the amplification. The expected amplicon sizes for each *Plasmodium* species are detailed in Table 3.

**Table 1:** Cycling conditions for primary PCR products (Nest 1)

Step	Temp	Time	Cycle
Initial denaturation	95 °C	5 min	1
Denaturation	95 °C	1 min	25x
Annealing,	60 °C	2 min	
Extension	72 °C	2 min	
Final annealing	60 °C	2 min	1
Final extension	72 °C	5 min	1
Hold	10 °C	∞

**Table 2:** Cycling conditions for second PCR products (Nest 2)

Step	Temp	Time	Cycle
Initial denaturation	95 °C	5 min	1
Denaturation	95 °C	1 min	30x
Annealing,	60 °C	2 min	
Extension	72 °C	2 min	
Final annealing	60 °C	2 min	1
Final extension	72 °C	5 min	1
Hold	10 °C	∞

**Table 3:** Primer sequences and PCR product sizes for Genus and Species-specific detection of *Plasmodium*

Genus/species	Primer name	Primer sequence	PCR product (bp)
*Plasmodium*	rPLU6	TCAAAGATTAAGCCATGCAAGTGA	1.6–1.7 kbp
	rPLU5	CCTGTTGTTGCCTTAAACTTC	
*P. falciparum*	rFAL1	TTAAACTGGTTTGGGAAAACCAAATATATT	206
	rFAL2	ACACAATGAACTCAATCATGACTACCCGTC	
*P. vivax*	rVIV1	CGCTTCTAGCTTAATCCACATAACTGATAC	121
	rVIV2	ACTTCCAAGCCGAAGCAAAGAAAGTCCTTA	
*P. malariae*	rMAL1	ATAACATAGTTGTACGTTAAGAATAACCGC	145
	rMAL2	AAAATTCCCATGCATAAAAAATTATACAAA	
*P. ovale*	rOVA1	ATCTCTTTTGCTATTTTTTAGTATTGGAGA	226
	rOVA2	ATCTAAGAATTTCACCTCTGACATCTG	

### Microscopic examination

Blood smears were stained with 10% buffer-diluted Giemsa stain and examined using a 100X oil immersion objective. Both thick and thin films were prepared for malaria parasite detection using the standard WHO slide preparation template. The three-reader method by WHO was employed [27]. Two mid-level microscopists (routine microscopy) independently examined and identified malaria parasites to the species level and performed parasite density counts in a blinded manner. We defined a mid-level microscopist as a qualified individual trained and certified by the Malaria Elimination Control Program of Ghana Health Service. Discordant results were assessed by a WHO-Certified Level 1 Expert microscopist, following the application of the Obare method calculator to determine if adjudication was warranted [31]. The reported microscopy dataset, referred to as adjudicated microscopy, represents the finalized results of the adjudication process, including concordant cases established through detection agreement with averaged counts from the two primary readers, and discordant cases resolved by expert review. A WHO-certified expert microscopist (Level 1) is an individual who demonstrates the highest competency in malaria microscopy, including accurate species identification and parasite quantification, validated through WHO proficiency testing [32]. Quality control, including testing malaria reagents with known positive and negative samples upon procurement of new reagents, was conducted at every stage of the process.

Malaria detection and quantification were analyzed and interpreted as follows: samples were reported as positive when malaria parasites were detected after scanning 100 fields on thick films; samples were reported as negative when no malaria parasites were seen, corresponding to “0” malaria parasites (mps) per 100 oil-immersion fields examined. Parasite infections reported included asexual forms in either single or mixed species infections. Parasite species reported included *Plasmodium falciparum* (Pf), *Plasmodium ovale* (Po), *Plasmodium vivax* (Pv), and *Plasmodium malariae* (Pm). Developmental stages of the parasite (trophozoites, schizonts, and gametocytes) were noted for reference but were not considered in the evaluation of miLab^™^ performance.

### Performing a parasite count and calculating parasite density

If malaria parasites were identified using thin films, quantification involved counting both parasitized and non-parasitized RBCs until a total of 2,000–5,000 RBCs was reached. The absolute method was used to determine parasite density [26].

#### Thin film count

For the thin film count, the formula for determining parasite density (*Parasite/µL*) was:

$${{Parasite} \mathord{\left/ {\vphantom {{Parasite} {\mu L}}} \right. \kern-0pt} {\mu L}}\, = \,\frac{Count\,of\,parasitized\,RBCs}{{Total\,RBC\,Count}}\, \times \,5,000,000$$

The total RBC count was the sum of parasitized and non-parasitized RBCs.

Additionally, as part of the parameters, we calculated the percentage of parasitized RBCs:

$$Parasitized\,RBC\,\% \, = \,\frac{Count\,of\,parasitized\,RBCs}{{Total\,RBC\,Count}}\, \times \,100$$

#### Thick film count

For malaria parasite count using thick films, the parasites were counted against White Blood Cells (WBCs). An estimated 200 to 500 WBCs were counted against parasites seen. The formula for determining parasite density using the thick smear was:

$$\frac{Parasite}{{\mu L}}\, = \,\frac{Count\,of\,parasites}{{Total\,WBC\,Counted}}\, \times \,{{8000\,white\,cells} \mathord{\left/ {\vphantom {{8000\,white\,cells} {\mu L}}} \right. \kern-0pt} {\mu L}}$$

The total WBC count was the sum of WBCs counted within 100 fields, with the total count not less than 200 WBCs. A standard value of 8000 white cells/µL was used.

### miLab^™^ and adjudicated microscopy against nPCR

The diagnostic performance of miLab^™^ was evaluated against adjudicated microscopy, with nPCR as the reference standard, where adjudicated microscopy refers to the finalized results of the adjudication process. While nPCR provides the analytical benchmark, relative comparisons with adjudicated microscopy and mid-level microscopists demonstrate miLab^™^’s operational feasibility, showing how it would perform in real-world clinical and field settings alongside standard malaria microscopy.

### Data analysis

Data was coded and entered using Microsoft Excel 2016 (Microsoft Corp., WA, USA), then exported to the GraphPad Prism Version 8.0.2 (Build: 263) for analysis. Descriptive statistics were computed to summarize the demographics of participants. Normality of continuous data was assessed using the Shapiro–Wilk test to determine the appropriateness of parametric or non-parametric methods.

miLab^™^ was evaluated at multiple assessment points. Its performance was assessed against nPCR, the reference standard, and in addition compared to routine microscopy (mid-level evaluations), which serves as the reference standard in clinical settings. To determine miLab^™^’s accuracy relative to microscopy, microscopy results were adjudicated according to WHO [27] and the Obare method calculator [31] to resolve discordant readings. Final comparisons to miLab^™^ were made using adjudicated microscopy results (expert microscopy), with nPCR as the reference standard.

Diagnostic accuracy parameters, sensitivity, specificity, Positive Predictive Value (PPV), Negative Predictive Value (NPV), and overall accuracy, were calculated with 95% confidence intervals using the Wilson-Brown method. Fisher’s exact test assessed statistical significance. Agreement between diagnostic methods was quantified using Cohen’s Kappa statistic and interpreted per [33] scale: values ≤ 0.20 indicated poor agreement, 0.21–0.39 minimal, 0.40–0.59 weak, 0.60–0.79 moderate, 0.80–0.90 strong, and ≥ 0.91 almost perfect agreement.

Associations between parasite densities from miLab™ and adjudicated microscopy were assessed using Spearman’s rank correlation coefficient, interpreted using thresholds from Chan: [34] poor (r < 0.30), fair (r = 0.30–0.50), moderately strong (r = 0.60–0.80), and very strong (r > 0.80). Bland–Altman analysis evaluated agreement in parasite quantification between methods. The analysis evaluated the mean difference and average parasite density between the two methods. A clinically significant threshold of ± 0.6 log parasites/μL was applied, consistent with Yekayo et al. [35] to minimize the risk of overinterpreting marginal discrepancies. Visualizations for parasite densities were all log transformed. All statistical tests were two-sided, and significance was set at p < 0.05 with a 95% confidence interval.

## Results

A total of 300 blood samples were analyzed using miLab^™^, adjudicated microscopy, and nPCR, the latter serving as the reference standard. Microscopy readings were performed independently by two mid-level microscopists (routine microscopy), with discrepancies resolved through adjudication to establish a reference standard. Subsequent analyses evaluated miLab^™^ performance against nPCR and adjudicated microscopy (expert microscopy). The performance of individual mid-level microscopists were also compared to nPCR.

### Participant characteristics

Of the 300 participants recruited for the study, majority were females 168 (56.00%) compared to males 132 (44.00%), aged 1–87 years, with a median age of 24 years (IQR: 15–43 years). Most of the participants were in the ≥ 15 years group (229, 76.33%), while children ≤ 5 years (38, 12.67%) and those aged 6–14 years (33, 11.00%) formed much smaller proportions (see Table 4).

**Table 4:** Characteristics of participants recruited for the study

Characteristic	Frequency, n (300)	Percentage
Gender		
Male	132	44.00
Female	168	56.00
Age		
≤ 5	38	12.67
6–14	33	11.00
≥ 15	229	76.33
Education		
No formal education	22	7.33
Basic level	58	19.33
Junior High level	35	11.67
Senior High level	55	18.33
Technical level	15	5.00
Tertiary level	109	36.33
Postgraduate level	6	2.00

### Feasibility study

A total of 30 blood samples were analyzed during the experimental phase, comprising 11 true positives and 18 true negatives as determined by nPCR. The miLab^™^ platform demonstrated excellent diagnostic performance, achieving 100% sensitivity and 94.74% specificity, with a Cohen’s kappa coefficient of 0.93, indicating near-perfect agreement with nPCR results. In comparison, WHO1 and WHO2 microscopists evaluations showed sensitivities of 72.73% and 81.82%, respectively, and both maintained a specificity of 94.74%. Similarly, Mid-level microscopist recorded 100% sensitivity, 94.74% specificity, and a kappa coefficient of 0.93 (Additional Table 2). This was a single mid-level microscopist's performance. The lower sensitivity observed for WHO1 reflects normal inter-operator variability that can occur even among certified expert microscopists in practical assessments, and shows differences in sample interpretation inherent to individual readings.

### Parasite detection across the detection methods

Detection of malaria parasites by genus, species, and stage, was largely consistent across methods. nPCR detected *Plasmodium falciparum* in 104 out of 300 cases (34.67%). miLab^™^ identified parasites in 100 cases (33.33%), comprising 99 *P. falciparum* and *1 Plasmodium vivax*, with both trophozoite and gametocyte stages observed. Among miLab^™^ positive samples, 81 cases (27.00%) were trophozoites only, while 19 cases (6.33%) displayed both trophozoite and gametocyte stages. Adjudicated microscopy revealed detection in 93 cases (31.00%), all identified as *P. falciparum* in the trophozoite stage. Mid-level microscopist 1 detected malaria parasites in 91 cases (30.33%), all identified as *Plasmodium falciparum* trophozoites. Mid-level microscopist 2 detected 68 positive cases (22.67%), also all *P. falciparum* trophozoites (Table 5).

**Table 5:** Detection of malaria parasites by Genus, Species, and Stage across detection methods

Detection method	n (300)	Genus detected n (%)	Species identified n (%)	Stage detected n (%)
nPCR				
Positive	104	104 (34.67)	*P. falciparum*: 104 (34.67)	N/A
Negative	196	196 (64.33)	N/A	N/A
miLab^™^				
Positive	100	100 (33.33)	*P. falciparum*: 99 (33.30) ^#^ *P. vivax*: 1 (0.3) ^*^	Trophozoite: 81 (27.00) Trophozoite/Gametocyte: 19 (6.33) ^#^
Negative	200	200 (66.67)		N/A
Microscopy (Adjudicated)				
Positive	93	93 (31.00)	*P. falciparum*: 93 (31.00)	Trophozoite: 93 (31.00)
Negative	207	207 (69.00)	N/A	N/A
Mid-level 1				
Positive	91	91 (30.33)	*P. falciparum*: 91 (30.33)	Trophozoite: 91 (30.33)
Negative	209	209 (69.67)	N/A	N/A
Mid-level 2				
Positive	68	68 (22.67)	*P. falciparum*: 68 (22.67)	Trophozoite: 68 (22.67)
Negative	232	232 (77.33)	N/A	N/A

N/A indicates species and stage identification were not applicable for negative results and nPCR

**Fig. 6:**
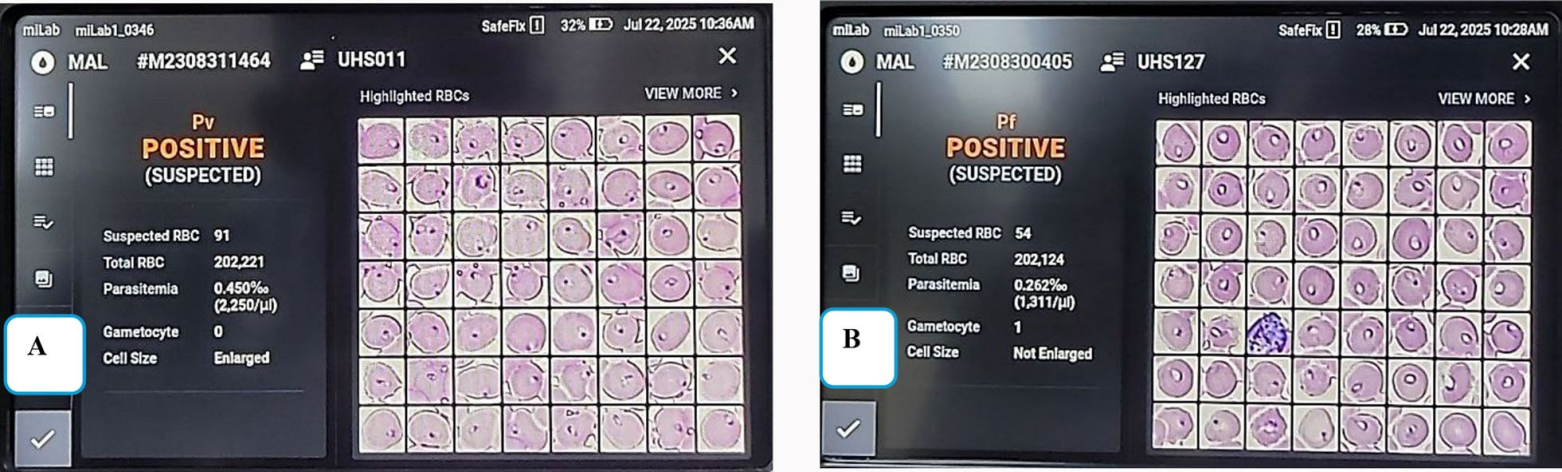
Screen display of a *P. vivax*-positive case **A** showing a total RBC count of 202,221 and a parasitemia level of 2,250/µL, and a *P. falciparum*-positive case **B** showing trophozoites with a gametocyte count of 1. All positive cases detected by miLab^™^ were predominantly at the trophozoite stage, with only a few cases presenting gametocytes as shown (Table 5). Trophozoites appear as ring forms within infected red blood cells, while the single gametocyte is identified by its characteristic crescent shape

^*^An image of *p. vivax* is provided in Fig. 6A

^#^An image of *p. falciparum*, trophozoite/gametocyte stage is shown in Fig. 6B

### Performance metrics

When evaluated against nPCR as the reference standard, miLab^™^ demonstrated high diagnostic performance, with a sensitivity of 94.23% (95% CI 87.98–97.33) and specificity of 98.98% (95% CI 96.36–99.82). Agreement was strong (κ = 0.94), and overall accuracy reached 97.33%. By comparison, adjudicated microscopy achieved lower sensitivity at 85.58% (95% CI 77.56–91.06) while maintaining high specificity (97.96%, 95% CI 94.87–99.20). Agreement (κ) and accuracy were high at 0.86 and 93.67% respectively. Comparison of mid-level 1 to nPCR revealed high sensitivity (87.50%, 95% CI 79.78–92.55) and specificity (100.00%, 95% CI 98.08–100.00), with strong agreement (κ = 0.90) and an overall diagnostic accuracy of 95.70%. For mid-level 2, however, sensitivity decreased to 59.62% (95% CI 50.01–68.54), maintaining a high specificity of 96.94% (95% CI 93.48–98.59), with moderate agreement (κ = 0.61), and overall accuracy of 84.00% (Table 6). Age-stratified performance metrics are available in Additional Table 3.

**Table 6:** Performance metrics of miLab™ and microscopy levels compared to nPCR

				Performance metrics					
				Sensitivity % (95% CI)	Specificity % (95% CI)	PPV % (95% CI)	NPV % (95% CI)	Kappa (%)	Accuracy (%)
		nPCR							
		Pos	Neg						
miLab^#^	Pos	98	2	94.23 (87.98–97.33)	98.98 (96.36–99.82)	98.00 (93.00–99.64)	97.00 (93.61–98.62)	94.10	97.33
	Neg	6	194						
		Pos	Neg						
Adjudicated microscopy^*^	Pos	89	4	85.58 (77.56–91.06)	97.96 (94.87–99.20)	95.70 (89.46–98.31)	92.75 (88.39–95.56)	85.70	93.67
	Neg	15	192						
		Pos	Neg						
Mid-level 1	Pos	91	0	87.50 (79.78–92.55)	100.00 (98.08–100.00)	100.00 (95.95–100.00)	93.78 (89.65–96.33)	90.01	95.70
	Neg	13	196						
		Pos	Neg						
Mid-level 2	Pos	62	6	59.62 (50.01–68.54)	96.94 (93.48–98.59)	91.18 (82.06–95.89)	81.90 (76.44–86.32)	61.60	84.00
	Neg	42	190						

Pos and Neg indicates Positive and Negative respectively

^#^Species detection for nPCR and miLab™ yielded 93.27% sensitivity, 98.98% specificity, kappa of 0.93 and accuracy of 97% due to the one *P. vivax* identified

^*^Species detection for nPCR and adjudicated microscopy attained 100% sensitivity and specificity for* P. falciparum*

### Parasite density distribution for miLab^™^ and microscopy

Of the 93 microscopy-positive cases confirmed by the WHO three-reader method, species identification was finalized using the third reader’s result where required. For parasite density estimation, WHO guidelines stipulate that the first two readers must independently agree on both positivity and species before counts are calculated. Discordant density estimates among such agreed positives require adjudication by a third reader. Applying this criterion, 61 cases proceeded to density analysis: 43 (70.49%) had discordant counts adjudicated by a WHO-certified expert using the Obare calculator, while 18 (29.51%) were concordant and their counts averaged. These finalized values served as the adjudicated microscopy densities for comparison with miLab™.

**Fig. 7:**
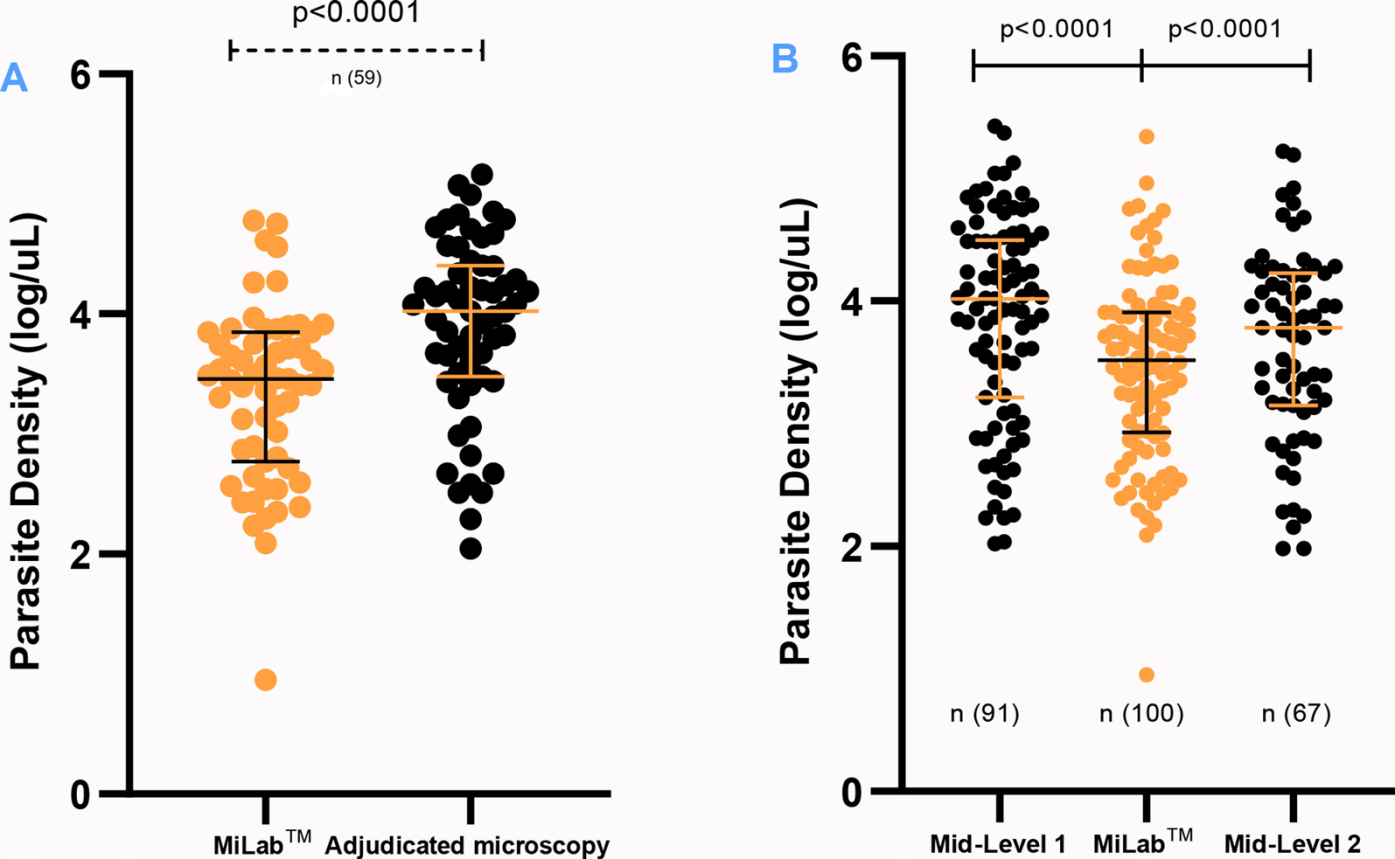
Log-transformed parasite density distribution between miLab^™^ and **A** adjudicated microscopy **B** mid-level microscopists. Each point represents an individual positive case. Horizontal bars indicate median values, and error bars represent the interquartile range (IQR: 25th–75th percentile)

Paired parasite density estimates (n = 59) showed that miLab^™^ ranged from 0.95 to 4.78 log parasites/µL (median = 3.46; IQR: 2.77–3.85), while adjudicated microscopy ranged from 2.05 to 5.16 log parasites/µL (median = 4.02; IQR: 3.48–4.40) (Fig. 7A). In Fig. 7B, parasite density estimates for positive cases across methods revealed miLab^™^ parasite densities ranged from a minimum of 0.95 to a maximum of 5.34, with a median of 3.52 (IQR: 2.93–3.91). For mid-level microscopist 1, densities ranged from 2.02 to 5.43, with a median of 4.02 (IQR: 3.22–4.50). Mid-level microscopist 2 measured densities between 1.98 and 5.22, with a median of 3.78 (IQR: 3.15–4.23).

### Correlation of parasite densities across detection methods

**Fig. 8:**
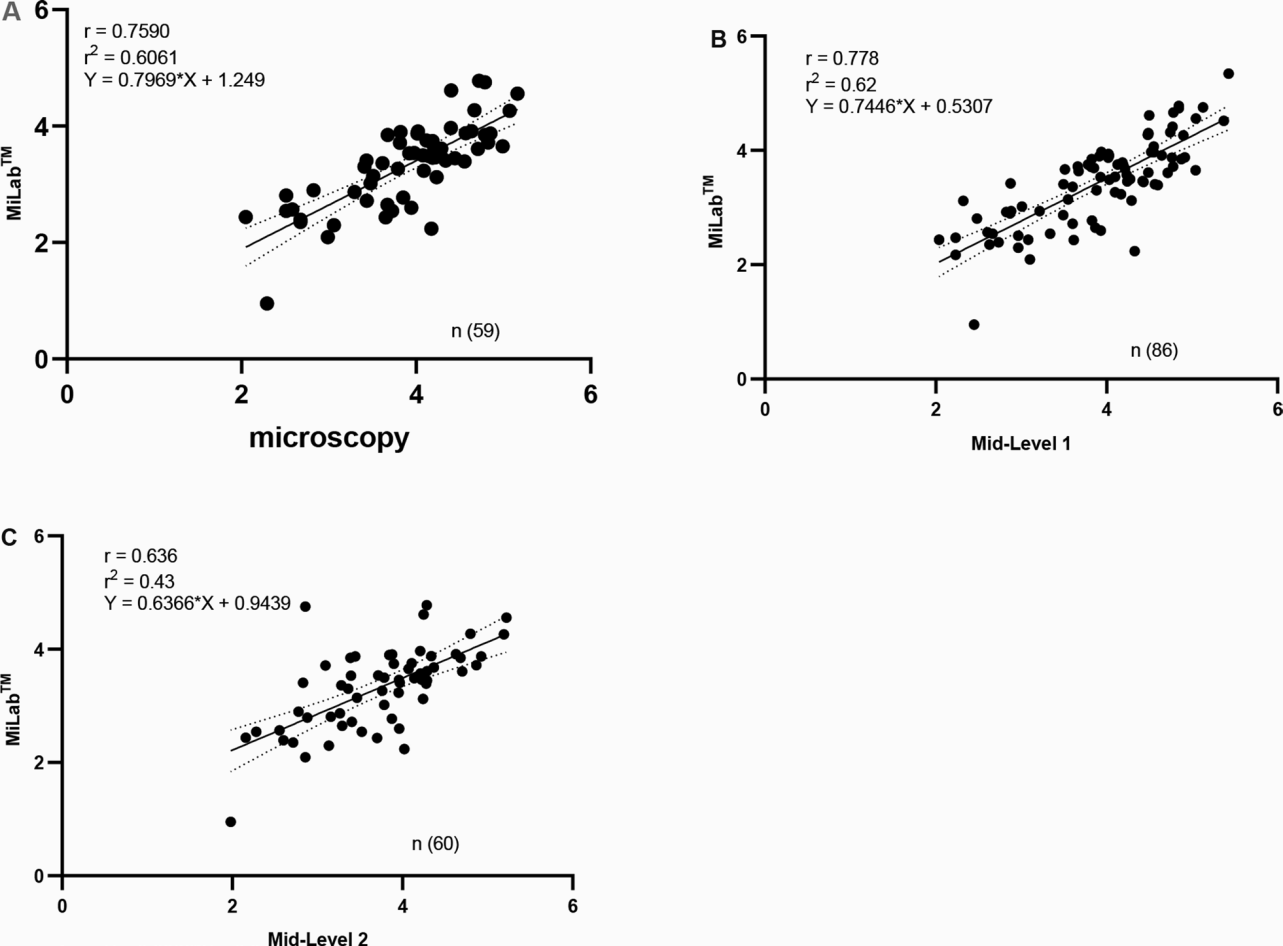
Correlation of parasite densities between miLab^™^ and **A** adjudicated microscopy **B** mid-level 1 microscopist and **C** mid-level 2 microscopist. Each point represents an individual positive case. The solid line indicates the line of best fit, and dotted lines represent the 95% confidence intervals

Correlation analyses as illustrated in Fig. 8A exhibits a moderately strong correlation of parasite densities for both miLab^™^ and adjudicated microscopy (Spearman’s r = 0.76, r^2^ = 0.61), while a moderately strong correlation for both miLab^™^ with mid-level 1 microscopist (Spearman’s r = 0.77, r^2^ = 0.62) (Fig. 8B) and mid-level 2 microscopist (Spearman’s r = 0.63, r^2^ = 0.43) (Fig. 8C) are displayed.

### Bland Altman analysis

**Fig. 9:**
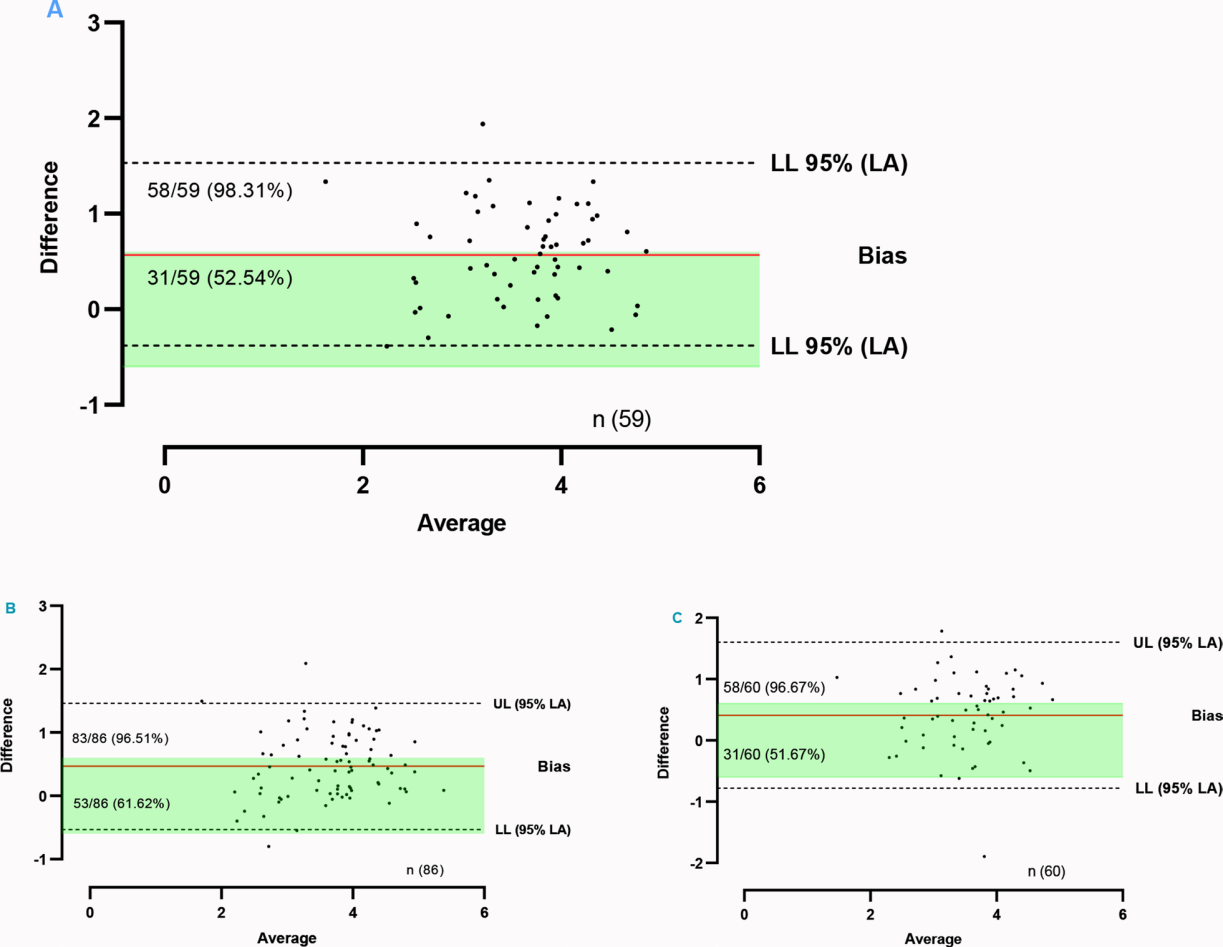
Parasite density agreement between miLab^™^ and **A** adjudicated microscopy **B** mid-level 1 microscopist and **C** mid-level 2 microscopist. Each point represents the difference between methods plotted against their mean parasite density (log-transformed). The solid red and dotted lines indicate the mean bias and 95% limit of agreement (LA) about the mean respectively, with LL depicting lower limit and UL as upper limit. Shaded regions show the clinically acceptable threshold defined by Yekayo, Fatoumata [35]

We conducted a Bland–Altman analysis to assess the level of agreement between miLab^™^ and microscopy levels by evaluating their mean difference and average parasite density readings. A clinically significant threshold of ± 0.6 log parasites/μL was adopted, aligning with Yekayo, Fatoumata [35]. miLab^™^, on average, recorded 0.57 units lower than adjudicated microscopy (Bias: 0.57). A total of 58 out of 59 paired observations (98.31%) fell within the 95% limits of agreement (CI −0.38 to 1.53), while 31/59 (52.54%) fell within the predefined clinically acceptable threshold (Fig. 9A). For mid-level 1 against miLab^™^, the mean bias was 0.47 log parasites/µL, with 95% limits of agreement ranging from −0.53 to 1.46. A total of 83/86 (96.51%) of data points fell within the limits of agreement, and 53/86 (61.62%) were within the clinically acceptable threshold (Fig. 9B). For mid-level 2 against miLab^™^, the mean bias was 0.41 log parasites/µL, with 95% limits of agreement from −0.78 to 1.60. A total of 58/60 (96.67%) of data points fell within these limits, and 31/60 (51.67%) were within the clinically acceptable threshold (Fig. 8C).

## Discussion

Malaria diagnosis remains central to effective clinical case management and epidemiological surveillance, yet its accuracy is often constrained by the skill of the microscopist, variability in slide preparation, and the inherent limitations of RDTs [15, 16]. Conventional microscopy remains the diagnostic gold standard in many endemic settings, but its performance is influenced by operator expertise, parasite density, and laboratory resources [15]. Molecular methods, including nPCR and quantitative PCR (qPCR), offer superior sensitivity and specificity but are impractical for routine use in most field environments due to cost, infrastructure, and turnaround time constraints [12].

Recent advances in automated microscopy platforms have sought to bridge this gap by integrating AI-driven parasite recognition algorithms with standardized staining techniques, thereby reducing inter-operator variability and improving throughput [17, 21–23]. Such platforms offer the potential to deliver near–expert-level performance in real time, even in resource-limited settings. However, their adoption requires rigorous evaluation across diverse diagnostic contexts, both against routine microscopy, which reflects real-world operational standards, and against adjudicated expert microscopy and molecular methods, which represent the highest achievable reference accuracy.

In this study, we benchmarked miLab^™^ against nPCR and achieved a high sensitivity and specificity, both exceeding 94.00% (Table 6), indicating excellent agreement with the molecular reference standard for detecting *Plasmodium* infections. At the species level, performance for *P. falciparum*, was similarly strong, with a sensitivity and specificity exceeding 93.00%, correctly identifying *P. falciparum* in 97 of 98 *P. falciparum* infections (Table 5). These results place miLab^™^ among the highest-performing automated platforms evaluated against molecular references, supporting its use for accurate case detection in moderate-to-high transmission settings. Comparable findings were reported by Enwetu et al. [22] who assessed miLab^™^ in Gondar (Ethiopia) and Kumasi (Ghana), obtaining sensitivities of 94.3% and 97.00% and specificities of 94.00% and 97.60% for *P. falciparum* and *P. vivax*, respectively. Similar molecular-level parity has been achieved by other automated systems, such as Parasight evaluated in India and Kenya, which reached 97.00% sensitivity and 95.00% specificity for *P. falciparum* against PCR [24]. Torres et al. [17] found Autoscope’s sensitivity and specificity against qPCR in San Juan Clinic, Peru (all species) to be 72.00% and 85.00%, respectively. Prescott et al. [25] similarly reported variable outcomes for a field-deployed microscopy image-analysis device, with pooled sensitivity and specificity of 92.00% and 90.00%, respectively, but site-specific results ranging from 89%/70% (WHO55 test) to 100%/94% (Equatorial Guinea malaria survey). In Southeast Asia, Delahunt et al. [23] reported that Autoscope achieved 100.00% sensitivity and 95.00% specificity on WHO55 evaluation slides for *P. falciparum* at parasite densities below 100 parasites/µL in Thailand. In the Netherlands, an automated microscopy system recorded 75.00% sensitivity and 99.90% specificity [20], while in Korea, Yoon et al. [21] found 100.00% sensitivity and specificity for both *P. vivax* and *P. falciparum* respectively.

Residual discordance between miLab^™^ and nPCR at genus level (FN = 6, FP = 2) and species level (FN = 7, FP = 2) is primarily attributable to differences in analytical sensitivity (Table 6). nPCR can detect submicroscopic infections at parasite densities below 5 parasites/µL [16], whereas most automated microscopy platforms operate at detection limits of 50–100 parasites/µL [21, 36]. For miLab^™^, the minimum detection threshold in this study was 9 parasites/µL (0.95 log), which is markedly lower than the operational limits of many comparable platforms [37, 38], yet infections below this threshold, particularly those < 5 parasites/µL, remain undetected when benchmarked against molecular methods. Like other automated image-analysis platforms, miLab^™^ performance is influenced by parasite species composition and mixed infections, as recognition algorithms are most effective for morphologically distinct *Plasmodium* species at moderate densities. Low-density non-*falciparum* and mixed-species infections are more likely to yield PCR-positive but microscopy-negative results [17]. The choice of reference standards also impact reported accuracy. PCR-based benchmarks consistently produce lower sensitivity estimates for microscopy [37]. In automated microscopy, factors such as image capture quality, smear preparation, and algorithm training datasets are critical to sensitivity. Suboptimal smear thickness, uneven Giemsa staining, or artefacts can lower classification confidence even above the nominal detection limit [17, 23]. Further inspection of the six nPCR-positive but miLab^™^-negative samples revealed that several had very low parasite densities by adjudicated microscopy (e.g., UHS125 = 124 parasites/µL; UHS131 = 192 parasites/µL; UHS108 = 64 parasites/µL), consistent with expected non-detection at the lower limit of sensitivity. However, discordance was also observed in cases with moderate or high parasite densities (e.g., UHS019 = 1,080 parasites/µL; UHS112 = 12,760 parasites/µL; UHS004 = 192 parasites/µL). These findings suggest that while most missed cases are attributable to low parasite densities, isolated discrepancies at higher loads likely reflect technical artefacts in smear preparation, atypical parasite morphology, or algorithm classification errors. This buttresses the importance of continuous refinement of image-recognition algorithms and strict slide quality control to minimize false negatives. Unlike manual microscopy, operator input in miLab^™^ is restricted to slide loading and calibration; there is minimal opportunity for real-time human correction of algorithm misclassifications, a process shown to improve specificity in other field-deployed systems [39]. Consequently, the robustness of the image recognition model and stringent quality control of slides are decisive determinants of performance.

In this evaluation, miLab^™^ detected 100 positives compared to 93 by adjudicated microscopy (Table 5), corresponding to minimum detection thresholds of 9 parasites/µL (0.95 log) for miLab^™^ and 64 parasites/µL (1.80 log) for adjudicated microscopy. Only 19 (FN = 15, FP = 4) discordant results remained, underlining the reproducibility of miLab^™^’s automated image capture and analysis in approximating expert-level detection. The lower detection threshold of miLab^™^ surpasses the operational limits of many automated platforms such as Parasight (20 parasites/µL) [37] and others with thresholds of 50–100 parasites/µL [21, 36, 38]. This capability is supported by miLab^™^’s preset analysis of approximately 200,000 red blood cells per smear (with the capacity to scan up to 300,000 RBCs) compared to the 2,000–5,000 RBCs typically examined in manual microscopy, greatly improving the likelihood of detecting low-density infections.

The strong correlation between miLab^™^ and adjudicated microscopy (Spearman’s r = 0.76) indicates that parasite density estimates produced by the device closely track those obtained by expert human readers, reinforcing its potential utility as a reliable quantification tool. The observed mean underestimation of 0.57 log parasites/µL, slightly lower to 0.359 log parasites/µL reported by Hamid et al. [39] while indicative of a slight systematic bias towards lower parasite counts, remains within clinically acceptable margins as defined by the WHO malaria microscopy quality assurance framework [26, 27], which defines the highest proficiency standard as ≥ 50% of counts falling within ± 25% of the reference value. This degree of bias is unlikely to significantly affect clinical decision-making, especially in settings where treatment thresholds are broad and prioritize detection over exact quantification. Furthermore, the Bland–Altman analysis demonstrates that > 98% of miLab™ results fall within the 95% limits of agreement, exhibiting strong concordance. However, approximately 53.00% of results were within the pre-defined stricter threshold of ± 0.6 log parasites/µL [35]. While this indicates room for improvement in precision, particularly for borderline cases or research contexts requiring high-fidelity quantification, the device’s performance remains operationally sound for routine diagnosis and monitoring.

Again, compared with nPCR, miLab^™^ demonstrated strong diagnostic performance, with a sensitivity of 94.23% and specificity of 98.98% (Table 6). The parasite detection performance of miLab^™^ is comparable to WHO Level 1 slide-panel accreditation, representing the highest sensitivity standard, whereas the adjudicated microscopy compared to the same reference standard more closely aligns with WHO Level 2, proficient at detecting parasite densities ≥ 100 parasites/µL but less sensitive at lower levels. This difference in analytical sensitivity likely accounts for the extra low-parasitemia detections by miLab^™^. This performance compares with findings from Ewnetu et al. [22] who noted sensitivities of > 94.00% and specificities of > 97.00% for miLab^™^ in a multi-country study. In Sudan’s high-transmission, performance of miLab^™^ with expert microscopy increased from substantial (κ = 0.65) to near perfect (κ = 0.97) in corrected mode [39].

Contrasting manual microscopy, miLab^™^’s algorithmic processing eliminates observer fatigue and inter-operator variability, maintaining reproducibility across high sample volumes. Nonetheless, optimal performance remains dependent on well-prepared and well-stained smears; artefacts, uneven film thickness, or staining deficiencies can reduce algorithm confidence despite adequate parasitemia. The high performance between miLab^™^ and adjudicated microscopy in this evaluation is comparable to, and in some cases exceeds, other AI-assisted microscopy platforms tested globally. Autoscope, for example, has shown sensitivity variability across settings, 72.00% in Peru’s San Juan Clinic but only 52.00% in Santa Clara, illustrating the impact of parasite density, slide quality, and adherence to design assumptions [17]. AI-assisted systems evaluated on WHO55 slides have achieved > 95.00% agreement with expert readers [40]. In this context, miLab™’s combination of high sensitivity, robust quantification agreement, low operational detection threshold, and minimal residual discordance positions it among the most capable automated microscopy systems currently available for malaria diagnosis.

Taken together, these findings suggest that miLab^™^ is highly consistent with adjudicated (expert) microscopy in parasite quantification, with a predictable and clinically tolerable downward bias. Its automated, standardized approach could reduce inter-reader variability inherent to manual microscopy, while maintaining adherence to WHO-recommended quality benchmarks. In high-burden or resource-limited settings where expert microscopists are scarce, such performance could represent a substantial advance in diagnostic capacity, particularly when coupled with its rapid turnaround time and reduced reliance on extensive training.

When compared under routine operational conditions, miLab^™^ showed strong concordance with mid-level microscopists in both parasite detection and quantification (Table 5, Figs. 7B and 9B, C). For the first mid-level microscopist, miLab^™^ produced parasite density estimates with a mean bias of 0.47 log parasites/µL, and 95% limits of agreement spanning −0.53 to 1.46. Notably, 96.50% of paired counts fell within these statistical limits, and 61.60% were within the pre-defined clinically acceptable range of ± 0.6 log parasites/µL. This indicates that, under real-world laboratory conditions, miLab^™^ yields parasite density measurements that are both statistically and clinically aligned with those from trained mid-level readers. Against the second mid-level microscopist, the mean bias was slightly lower at 0.41 log parasites/µL, although the limits of agreement were somewhat wider (−0.78 to 1.60 log parasites/µL). Even so, 96.70% of results fell within statistical limits and 51.70% within the clinically acceptable threshold. Such variation likely reflects well-recognized sources of imprecision in field microscopy, subtle inconsistencies in smear preparation, staining quality, and parasite counting technique [41]. The mean biases observed indicate a modest systematic overestimation of parasite density. Interpreted within WHO’s malaria microscopy quality framework [26, 27], these differences fall within an acceptable operational range for clinical use. This level of performance supports the utility of miLab™ for guiding treatment decisions, including in severe malaria, and for monitoring therapeutic response, while its reproducibility across multiple microscopists shows its potential to reduce inter-operator variability, a persistent limitation of routine malaria microscopy.

The qualitative detection performance mirrored these quantitative findings (Table 6). When evaluated against nPCR, miLab^™^ achieved a high sensitivity and specificity consistently outperforming mid-level readers. While one reader showed strong concordance with nPCR, performance varied notably for the second reader, illustrating the well-documented issue of inter-operator variability in malaria microscopy, particularly in sensitivity at low parasite densities [41]. In this context, miLab^™^’s performance reflects its potential to minimize subjectivity and ensure reproducibility across different users and settings. This level of diagnostic performance closely aligns with, Hamid et al. [39] who reported miLab^™^ sensitivities and specificities exceeding 93%, in a Sudanese point-of-care study, weighing the platform’s consistent performance across different geographic and operational contexts. This results also aligns with performance of other AI-assisted systems in operational contexts, such as Autoscope’s 95–98% sensitivity and 90–95% specificity in Thailand and Peru [17, 23]. Collectively, these findings support miLab^™^ as a robust complement to routine malaria microscopy, providing reliable support for clinical decision-making and therapeutic monitoring, while mitigating the inter-operator variability that frequently undermines diagnostic consistency in field practice.

## Conclusion

Across both operational and high-precision analytical contexts, miLab^™^ MAL demonstrated robust diagnostic performance for malaria detection and parasite quantification. Under routine laboratory conditions with mid-level microscopists, the platform achieved high concordance in both qualitative and quantitative measures, with more than half parasite density counts within clinically acceptable thresholds. Against expert microscopy and nPCR, miLab^™^ maintained strong sensitivity and specificity, with reduced bias and narrower limits of agreement, indicating high analytical precision. These findings are aligned with the strategic objectives of Ghana’s National Malaria Elimination Programme (NMEP) framework, which emphasizes strengthening diagnostic accuracy, ensuring quality-assured microscopy, and integrating innovative technologies to support case management and surveillance. miLab^™^’s reproducibility across different operator skill levels, combined with its compatibility with reference-grade benchmarks, positions it as an essential adjunct to existing diagnostic infrastructure. Its adoption could enhance both routine case detection and the generation of high-quality epidemiological data, supporting Ghana’s efforts to accelerate malaria elimination while maintaining alignment with global malaria control and elimination goals.

### Limitations

The study included limited *P. vivax* and other non-*falciparum* species, which restricts conclusions regarding cross-species diagnostic accuracy in mixed-endemic contexts.

## Supplementary Information

Additional file 1. Figure 1. miLab™ MAL diagnostic platform with cartridge

Additional file 2. Table 1. Detailed specifications of miLab™ MAL equipment.

Additional file 3. Table 2. Performance metrics of miLab™ compared with nested PCR during the feasibility study.

Additional file 4. Table 3. Age-stratified performance metrics of milab^TM^ against different diagnostic comparators.

## Data Availability

The datasets supporting the conclusions of this article are included within the article.
