# Supplementary figures and images for: Evaluating the diagnostic performance of miLab™ for detection of malaria parasites using nPCR as reference standard
Source: Malar J. 2026 Feb 12;25:109. doi: 10.1186/s12936-026-05801-7 (PMC12922333; doi:10.1186/s12936-026-05801-7)

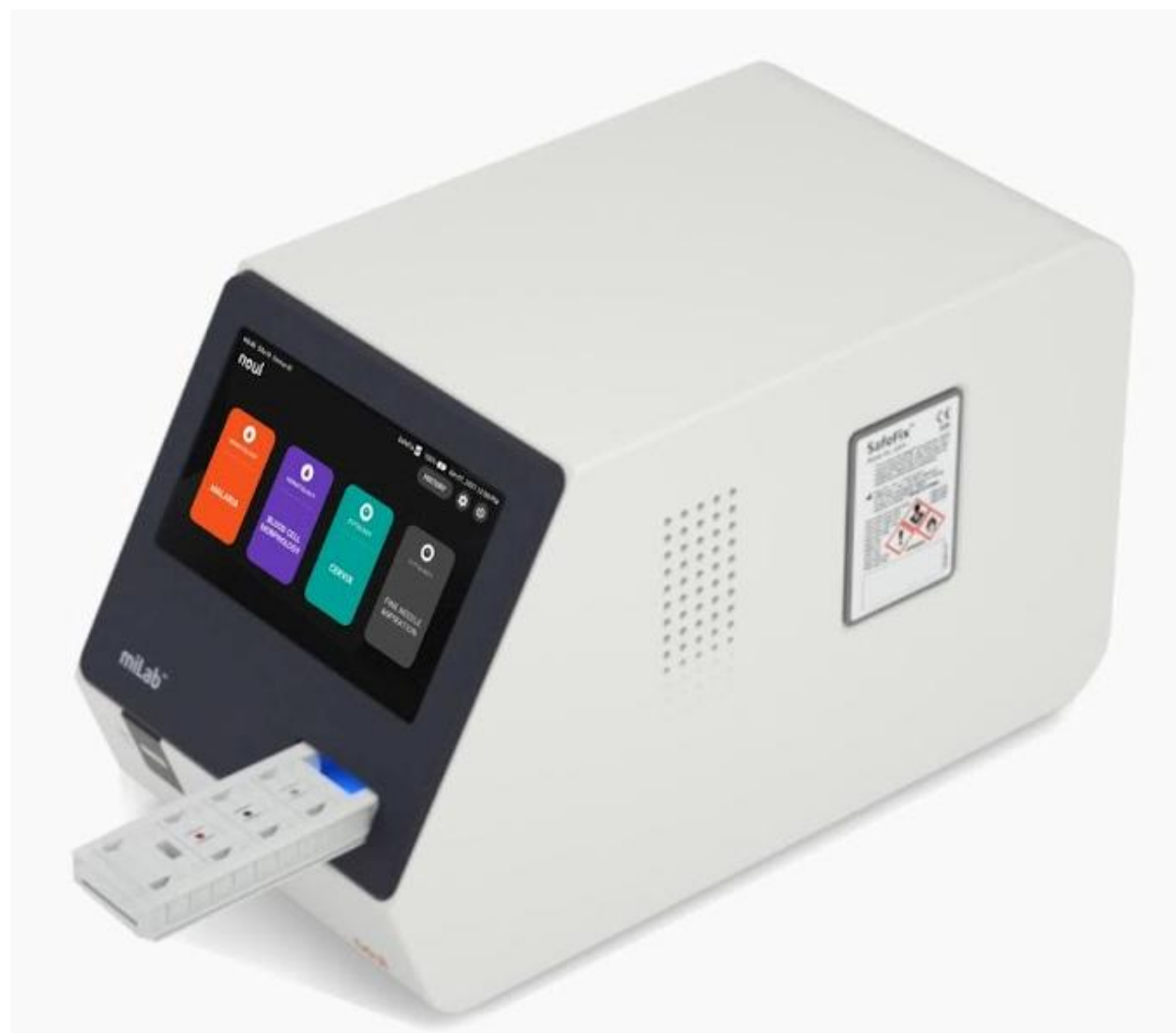

Supplement: Supplementary file 1 — Additional file 1. Figure 1. miLab™ MAL diagnostic platform with cartridge [file 12936_2026_5801_MOESM1_ESM.pdf]
