# Supplementary material for: Evaluating the diagnostic performance of miLab™ for detection of malaria parasites using nPCR as reference standard
Source: Malar J. 2026 Feb 12;25:109. doi: 10.1186/s12936-026-05801-7 (PMC12922333; doi:10.1186/s12936-026-05801-7)
Supplement: Supplementary file 2 — Additional file 2. Table 1. Detailed specifications of miLab™ MAL equipment. [file 12936_2026_5801_MOESM2_ESM.pdf]

Specification	Type	Description
**Sample Preparation**	Staining method	Romanowsky
	Sample type	Venous or finger prick
	Fixative	SafeFix
	Sample volume	5 µl
**Imaging**	Focusing	Multi-focus images acquired
	Image count	Acquires max. of 300,000 RBCS
**Analysis**	Species suggestions	*Plasmodium falciparum* (Pf)<br>*Plasmodium vivax* (Pv)<br>*Plasmodium* spp. (MP)
	Stages suggestions	Gametocyte, Schizont, Ring / Trophozoite
	Count	Suspected RBC, Total RBC
	Parasitemia level	% (Suspected RBC/Total RBC), Parasites/uL (assuming 5 million RBC/uL)
	Viewing functions	• Field view<br>• Multi-focus view (10 vertical-planes)
**Total Test Time**		Average 15 minutes
**Weight**		Approximately 11 kg
**Remote View**	Viewing functions	Field View, Cell View (10 images from each cell along the Z axis), Cell Reclassification, Report, 7.5 inches touch screen
**Battery**	Lifespan	3-4 hours
**Version**	System SW	v01.52.00
**Version**	SW	1.2.0
