# Supplementary material for: Evaluating the diagnostic performance of miLab™ for detection of malaria parasites using nPCR as reference standard
Source: Malar J. 2026 Feb 12;25:109. doi: 10.1186/s12936-026-05801-7 (PMC12922333; doi:10.1186/s12936-026-05801-7)
Supplement: Supplementary file 3 — Additional file 3. Table 2. Performance metrics of miLab™ compared with nested PCR during the feasibility study. [file 12936_2026_5801_MOESM3_ESM.pdf]

		nPCR		Sensitivity	Specificity	Kappa
		pos	neg			
**milab**	pos	11	1	100	94.74	93
	neg	0	18			
**WHO1**	pos	8	1	72.73	94.74	70.1
	neg	3	18			
**WHO2**	pos	9	1	81.82	94.74	78
	neg	2	18			
**ML 1**	pos	11	1	100	94.74	93
	neg	0	18			
