# Supplementary material for: Evaluating the diagnostic performance of miLab™ for detection of malaria parasites using nPCR as reference standard
Source: Malar J. 2026 Feb 12;25:109. doi: 10.1186/s12936-026-05801-7 (PMC12922333; doi:10.1186/s12936-026-05801-7)
Supplement: Supplementary file 4 — Additional file 4. Table 3. Age-stratified performance metrics of milabTM against different diagnostic comparators. [file 12936_2026_5801_MOESM4_ESM.pdf]

Additional Table 2. Age-stratified performance metrics of milab against different diagnostic comparators

				Performance Metrics					
**Comparator vs miLab**				**Sensitivity % (95% CI)**	**Specificity % (95% CI)**	**PPV % (95% CI)**	**NPV % (95% CI)**	**Kappa (%)**	**Accuracy (%)**
**Age**	**miLab**	**nPCR**							
		**Pos**	**Neg**						
≤ 5	Pos	4	1	1.00 (0.51-1.00)	0.97 (0.85-1.00)	0.80 (0.38-0.99)	1.00 (0.90-1.00)	0.87	97.4
	Neg	0	33						
		**Mid-Level 1**							
	Pos	2	3	1.00 (0.18-1.00)	0.92 (0.78-0.97)	0.40 (0.071-0.77)	1.00 (0.90-1.00)	0.54	92.1
	Neg	0	33						
		**Mid-Level 2**							
	Pos	2	3	1.00 (0.18-1.00)	0.92 (0.78-0.97)	0.40 (0.071-0.77)	1.00 (0.90-1.00)	0.54	92.1
	Neg	0	33						
		**Adjudicated Microscopy**							
	Pos	2	3	1.00 (0.18-1.00)	0.92 (0.78-0.97)	0.40 (0.071-0.77)	1.00 (0.90-1.00)	0.54	92.1
	Neg	0	33						
6-14		**nPCR**							
		**Pos**	**Neg**						
	Pos	10	0	1.00 (0.72-1.00)	1.00 (0.86-1.00)	1.00 (0.72-1.00)	1.00 (0.86-1.00)	1.00	100
	Neg	0	23						
		**Mid-Level 1**							
	Pos	10	0	1.00 (0.72-1.00)	1.00 (0.86-1.00)	1.00 (0.72-1.00)	1.00 (0.86-1.00)	1.00	100
	Neg	0	23						
		**Mid-Level 2**							
	Pos	7	3	0.88	0.88	0.70	0.96	0.70	87.9

	Neg	1	22	(0.53-0.99)	(0.70-0.93)	(0.40-0.89)	(0.79-1.00)		
		**Adjudicated Microscopy**							
	Pos	10	0	1.00 (0.72-1.00)	1.00 (0.86-1.00)	1.00 (0.72-1.00)	1.00 (0.86-1.00)	1.00	100
	Neg	0	23						
		**nPCR**							
**≥ 15**	Pos	84	1	0.93 (0.86-0.97)	0.99 (0.96-1.00)	0.99 (0.94-1.00)	0.96 (0.91-0.98)	0.94	96.9
	Neg	6	138						
		**Mid-Level 1**							
	Pos	74	11	0.94 (0.86-0.97)	0.93 (0.87-0.96)	0.87 (0.78-0.93)	0.97 (0.92-0.99)	0.85	93.0
	Neg	5	139						
		**Mid-Level 2**							
	Pos	51	34	0.88 (0.77-0.94)	0.80 (0.74-0.85)	0.60 (0.49-0.70)	0.95 (0.90-0.98)	0.59	82.1
	Neg	7	137						
		**Adjudicated Microscopy**							
	**Pos**	73	12	0.90 (0.82-0.95)	0.92 (0.86-0.95)	0.86 (0.77-0.92)	0.94 (0.89-0.97)	0.81	91.3
	**Neg**	8	136						

Pos and Neg indicates Positive and Negative respectively
